# Subtyping of Group 3/4 medulloblastoma as a potential prognostic biomarker among patients treated with reduced dose of craniospinal irradiation: a Japanese Pediatric Molecular Neuro-Oncology Group study

**DOI:** 10.1186/s40478-023-01652-4

**Published:** 2023-09-25

**Authors:** Kohei Fukuoka, Jun Kurihara, Tomoko Shofuda, Naoki Kagawa, Kai Yamasaki, Ryo Ando, Joji Ishida, Masayuki Kanamori, Atsufumi Kawamura, Young-Soo Park, Chikako Kiyotani, Takuya Akai, Dai Keino, Yosuke Miyairi, Atsushi Sasaki, Junko Hirato, Takeshi Inoue, Atsuko Nakazawa, Katsuyoshi Koh, Ryo Nishikawa, Isao Date, Motoo Nagane, Koichi Ichimura, Yonehiro Kanemura

**Affiliations:** 1 Department of Hematology/Oncology, Saitama Children’s Medical Center, 1-2, Shin-Toshin, Saitama, 330-8777 Japan; 2 Department of Neurosurgery, Saitama Children’s Medical Center, Saitama, Japan; 3 Department of Biomedical Research and Innovation, Institute for Clinical Research, Osaka National Hospital, National Hospital Organization, Osaka, Japan; 4 Department of Neurosurgery, Osaka University Graduate School of Medicine, Osaka, Japan; 5 Department of Pediatric Hematology and Oncology, Osaka City General Hospital, Osaka, Japan; 6 Department of Neurosurgery, Chiba Children’s Hospital, Chiba, Japan; 7 Department of Neurological Surgery, Okayama University Graduate School, Okayama, Japan; 8 Department of Neurosurgery, Tohoku University Graduate School of Medicine, Sendai, Japan; 9 Department of Neurosurgery, Hyogo Prefectural Kobe Children’s Hospital, Kobe, Japan; 10 Department of Neurosurgery, Nara Medical University, Kashihara, Japan; 11 Children’s Cancer Center, National Center for Child Health and Development, Tokyo, Japan; 12 Departments of Neurosurgery, Graduate School of Medicine and Pharmaceutical Science, University of Toyama, Toyama, Japan; 13 Division of Hematology/Oncology, Kanagawa Children’s Medical Center, Yokohama, Japan; 14 Department of Neurosurgery, Nagano Children’s Hospital, Azumino, Japan; 15 Department of Pathology, Saitama Medical University, Saitama, Japan; 16 Department of Pathology, Public Tomioka General Hospital, Gunma, Japan; 17 Department of Pathology, Osaka City General Hospital, Osaka, Japan; 18 Department of Clinical Research, Saitama Children’s Medical Center, Saitama, Japan; 19 Department of Neuro-Oncology/Neurosurgery, Saitama Medical University International Medical Center, Saitama, Japan; 20 Department of Neurosurgery, Kyorin University Faculty of Medicine, Mitaka, Japan; 21 Department of Brain Disease Translational Research, Juntendo University Faculty of Medicine, Tokyo, Japan

**Keywords:** Medulloblastoma, Methylation analysis, Prognostic stratification

## Abstract

**Background:**

One of the most significant challenges in patients with medulloblastoma is reducing the dose of craniospinal irradiation (CSI) to minimize neurological sequelae in survivors. Molecular characterization of patients receiving lower than standard dose of CSI therapy is important to facilitate further reduction of treatment burden.

**Methods:**

We conducted DNA methylation analysis using an Illumina Methylation EPIC array to investigate molecular prognostic markers in 38 patients with medulloblastoma who were registered in the Japan Pediatric Molecular Neuro-Oncology Group and treated with reduced-dose CSI.

**Results:**

Among the patients, 23 were classified as having a standard-risk and 15 as high-risk according to the classic classification based on tumor resection rate and presence of metastasis, respectively. The median follow-up period was 71.5 months (12.0–231.0). The median CSI dose was 18 Gy (15.0–24.0) in both groups, and 5 patients in the high-risk group received a CSI dose of 18.0 Gy. Molecular subgrouping revealed that the standard-risk cohort included 5 WNT, 2 SHH, and 16 Group 3/4 cases; all 15 patients in the high-risk cohort had Group 3/4 medulloblastoma. Among the patients with Group 3/4 medulloblastoma, 9 of the 31 Group 3/4 cases were subclassified as subclass II, III, and V, which were known to an association with poor prognosis according to the novel subtyping among the subgroups. Patients with poor prognostic subtype showed worse prognosis than that of others (5-year progression survival rate 90.4% vs. 22.2%; *p* < 0.0001). The result was replicated in the multivariate analysis (hazard ratio12.77, 95% confidence interval for hazard ratio 2.38–99.21, *p* value 0.0026 for progression-free survival, hazard ratio 5.02, 95% confidence interval for hazard ratio 1.03–29.11, *p* value 0.044 for overall survival).

**Conclusion:**

Although these findings require validation in a larger cohort, the present findings suggest that novel subtyping of Group 3/4 medulloblastoma may be a promising prognostic biomarker even among patients treated with lower-dose CSI than standard treatment.

**Supplementary Information:**

The online version contains supplementary material available at 10.1186/s40478-023-01652-4.

## Introduction

Reducing the dose of craniospinal irradiation (CSI) in patients with medulloblastoma to minimize neurological sequelae in survivors is challenging. For non-metastatic medulloblastomas, a large international prospective study failed to show the non-inferiority of reduced-dose craniospinal irradiation to standard treatment [1]. The presence of dissemination still indicates a high risk of recurrence, suggesting the need for high-dose CSI, leading to potentially severe neurocognitive complications even in this era of molecular classification [2, 3]. Although several promising biomarkers for finer prognostic stratification have recently been reported, including *MYC*/*MYCN* amplification [4, 5], whole chromosomal aberration signature [6], and molecular subtyping by DNA methylation profiling in Group 3/4 tumors [7], whether these molecular markers have the same clinical impact in patients treated with lower dose CSI (LD-CSI) than standard treatment remains unknown since these data were derived from analysis among patients receiving standard care. To date, there has been only one report by Michalski et al. on the molecular data of patients with medulloblastoma who received LD-CSI [1]. The authors revealed outcome differences depending on the presence of molecular alterations, including *TP53* mutation in SHH and loss of chromosome (chr) 11 in Group 4, but had no data on methylation subtyping analysis [1].

In Japan, LD-CSI has been performed for patients with medulloblastoma in clinical trials or at discretion of physicians since the 2010s, and the majority of patients have been in remission for a certain period [8, 9]. We recently reported a long-term survivor case series of metastatic medulloblastoma treated with 24 gray (Gy) of CSI that demonstrated the potential prognostic significance of novel subtyping in Group 3/4 [9]. To explore the prognostic implications, we conducted a retrospective clinico-molecular analysis of medulloblastomas using genome-wide DNA methylation analysis with a larger cohort and unveiled the clinical significance of subtyping Group 3/4 medulloblastoma as a promising prognostic biomarker for reducing CSI dose in patients with medulloblastoma.

## Materials and methods

### Tumor materials

We performed molecular analysis combined with morphological and clinical prognostic analysis on medulloblastomas cases registered into Japan Pediatric Molecular Neuro-oncology Group (JPMNG), which is combined project from both Japan Society of Neuro-Oncology and Japan Society of Pediatric Neurosurgery (Table 1). Among the registered patients in the project, we selected 38 patients who were treated with a lower dose of CSI than the standard of care from 1996 to 2018. This study was approved by the ethics committees of the institutions. Pathological diagnoses were performed according to 2016 World Health Organization (WHO) classification of Central Nervous System [10].

**Table 1:** Clinical and molecular characteristics of cases

Clinical risk	Average risk (Gross total resection/Metastasis(−))	High risk (Metastasis(+))
Case number (n)	23	15 (M1: 4 cases, M2: 5 cases, M3: 6 cases)
Age at onset (median, years)	6.9 (3.0–22.1)	6.8 (2.8–15.1)
Pathological subtype	Classic, 22	Classic, 14
	Desmoplastic/Nodular, 1	Large cell/Anaplastic, 1
Molecular subgroup (n)	WNT, 4	Group 3, 7
	SHH, 3	Group 4, 8
	Group 3, 2	
	Group 4, 14	
Group 3/4 subclass (n)	Subclass I, 1	Subclass II, 4
	Subclass IV, 1	Subclass III, 1
	Subclass V, 2	Subclass IV, 1
	Subclass VI, 1	Subclass V, 2
	Subclass VII, 7	Subclass VI, 1
	Subclass VIII, 4	Subclass VII, 5
		Subclass VIII, 1
Craniospinal irradiation dose (median, Gray)	18.0 (15–18)	24.0 (18–24)
Local irradiation dose (median, Gray)	50 (50–60)	50.4 (46–55.8)
Intrathecal chemotherapy (n)	Yes: No = 18: 4	Yes: No = 11: 3
	(unknown in one case)	(unknown in one case)
Observation period (median, months)	74	49.8
	(14.0–231.0)	(11.7–177.6)

### Genome-wide DNA methylation analysis and copy number analysis

We performed comprehensive methylation analysis of all tumors using the Illumina Infinium HumanMethylationEPIC (EPIC array) or HumanMethylation450k (450 k array) BeadChip array (Illumina, San Diego, CA, USA) which includes 866,238 or 485,512 CpG sites for analysis. Methods for t-distributed stochastic neighbor embedding (t-SNE) was described elsewhere [11]. The reference methylation data of 183 medulloblastomas (GSE90496) were obtained from the Gene Expression Omnibus database (http://www.ncbi.nlm.nih.gov/geo/) for comparison with our data. To get molecular classification data, a molecular classification algorithm from the German Cancer Center (DKFZ classifier, https://www.molecularneuropathology.org/mnp) was employed [12]. The classifier designates molecular “class” based on a comparison between observed methylation data and a reference cohort consisting of over 2800 neuropathological tumors of almost all known entities. We adopted the classifier result of version 12.5. When there was a discordance of the molecular subtype of Group3/4 medulloblastoma between tSNE plotting and the methylation classifier with a prediction score called “calibrated score” under 0.9, the former analysis result was adopted because the classifier defined results with the calibrated score above 0.9 reliable [12]. DNA copy number was calculated from raw signal intensities from methylation data obtained from the webpage of the German Cancer Center as described above.

### Definition of the prognostic biomarkers

Subtypes II, III, and V were determined as poor prognostic subtypes in Group 3/4 medulloblastomas due to the worse prognosis among subtypes in the past literature [7]. Whole chromosomal aberration (WCA) signatures were defined as the presence of at least two of three chromosomal changes as follows: chromosome (chr) 7 gain, chr 8 loss, and chr 11 loss [6].

### Statistical analysis

For statistical analysis, subgroup comparisons were performed by t-test, Pearson’s chi-square test, Fisher’s exact test, and Wilcoxon rank-sum test. Overall survival (OS) was defined as the probability of survival, with only death as the event. Progression-free survival (PFS) was defined as the probability of being alive and free of progression or relapse. Survival curves were plotted using the Kaplan–Meier method. The log-rank test and Cox proportional hazards model were used to detect differences in survival between different groups of patients. Two-sided tests were used for all analyses, and *p* values < 0.05 were considered significant. JMP 9 (SAS Institute Inc., Cary, NC, USA) was used for all analyses.

## Results

### Cohort clinical characteristics

A total of 38 patients were included in this analysis (Table 1, Additional file 1: Table). They consisted of 23 standard-risk (SR) and 15 high-risk (HR) patients (M1: 4cases, M2: 5 cases, M3: 6 cases) according to the clinical risk stratification consisting of resection rate and presence of metastasis [13]. All patients, except for one developed the disease at the age of 3 or older. The pathological subtype was the classic type in most cases. In terms of treatment, the median CSI dose was a median 18.0 (15.0–18.0) Gy in the SR group and 24.0 (18.0–24.0) Gy in the HR group. All SR patients except for one received 18.0 Gy of CSI. Among HR patients, 5 also received 18.0 Gy of CSI, and the rest of the patients were irradiated with 23.4 or 24.0 Gy of CSI to the whole neuroaxis. Local irradiation was performed at the focal tumor site in all cases, with a median dose of 50.0 (46.0–60.0) Gy. The majority of patients (29 out of 38 patients) received intrathecal chemotherapy involving six doses of methotrexate. Regarding the chemotherapeutic regimen, 92% (35 out of 38 patients) received treatment as per the Japan Pediatric Brain Tumor Consortium protocol [8], which includes four courses of cyclophosphamide, cisplatin, vincristine, and etoposide (Additional file 1: Table). In this protocol, radiation therapy was concurrently administered during the second and third courses of chemotherapy, and HR patients underwent high-dose chemotherapy. The median follow-up time was 74.0 months (14.0–231.0) in the SR and 49.8 months (11.7–177.6) in the HR group.

### Molecular characterization of medulloblastomas treated with lower dose CSI

**Fig. 1:**
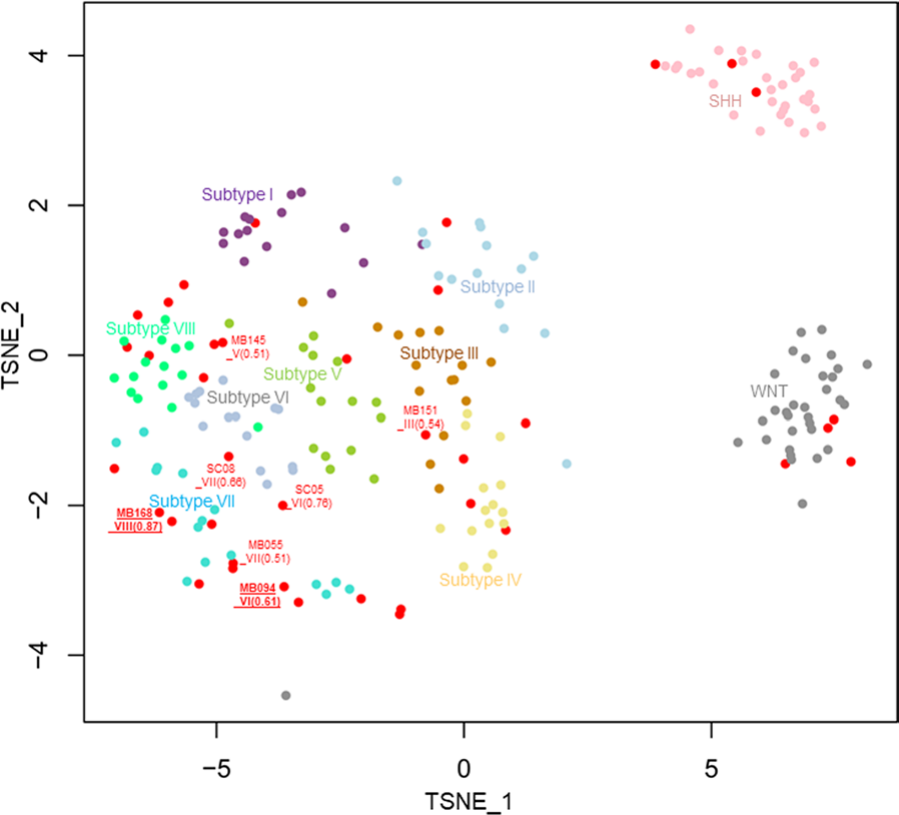
t-stochastic neighbor embedding (t-SNE) plot of methylation data from our cases and reference data of medulloblastomas. Cases in our cohort are highlighted in red in the tSNE plot. Cases that did not show a calibrated score of more than 0.9 were plotted with the case number, subtype number, and calibrated score

Regarding the molecular subgroup, there were four WNT-activated, three SHH-activated, two Group 3, and 14 Group 4 cases in the SR cohort, although no WNT or SHH cases were seen in the HR cohort. Among Group 3/4 tumors, most cases showed a calibrated score of 0.9 or higher for the subtyping from the methylation classifier v.12.5, and concordance between the classifier and tSNE analysis. Of the seven cases (18%) with calibrated scores under 0.9, two cases (5%) presented discordant subtyping results between the classifier and tSNE plotting (Fig. 1). They were classified as subtype VI or VIII by the classifier but clustered with subtype VII in the tSNE analysis (Fig. 1).

**Fig. 2:**
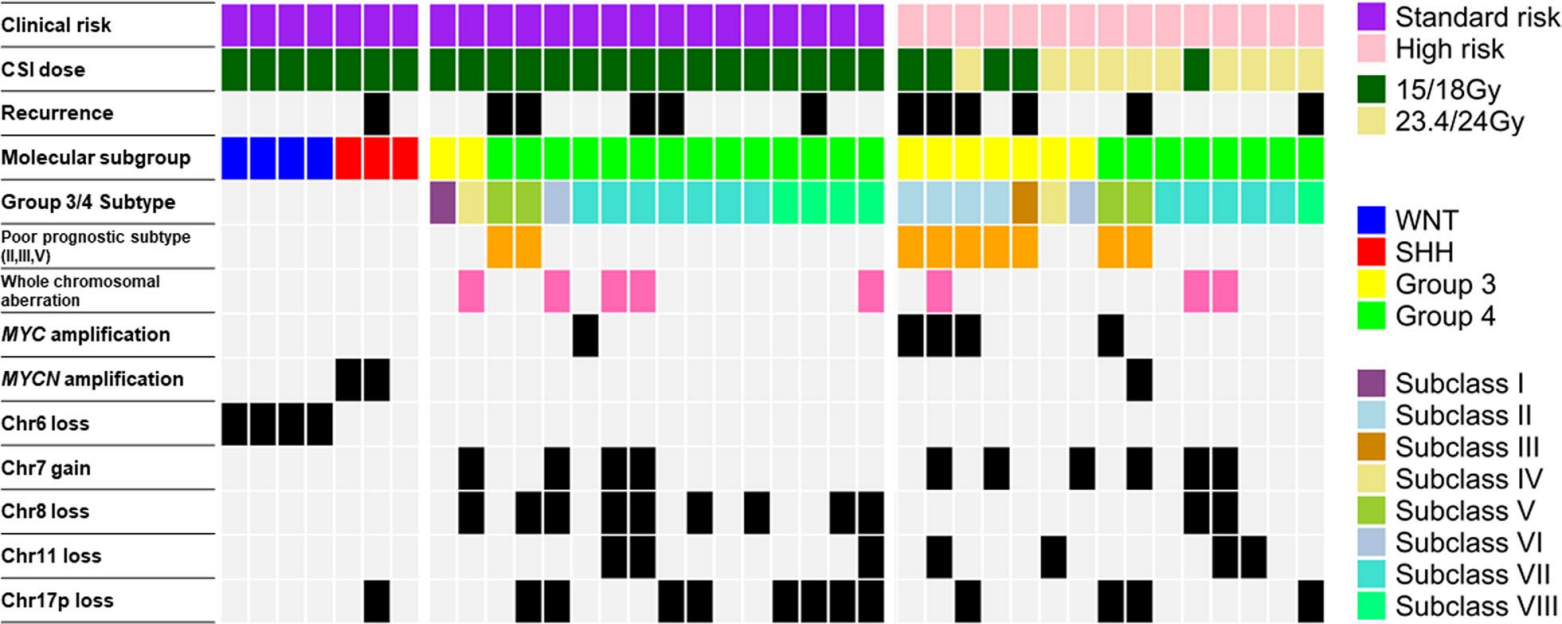
Epigenetic, clinical, and molecular features of the study cohort described using an oncoprint. Clinical risk is indicated in the top column. The other column indicates CSI dose, Molecular subgrouping, Group 3 or 4 subtyping, prognostic subtype, presence of whole chromosomal aberration, and presence of genetic alterations, including *MYC* amplification, *MYCN* amplification, chr6 loss, chr7 gain, chr8 loss, chr 11 loss, and chr 17p loss

In terms of copy number abnormalities (Fig. 2), all WNT cases showed chr 6 loss, and two out of three SHH tumors exhibited *MYCN* amplification. WCA signatures were observed in five SR cases and three HR cases, and one SR case and five HR cases showed *MYC* or *MYCN* amplification among Group 3/4 tumors. Regarding Group 3/4 subtyping (Fig. 2), only two SR cases were included in the poor prognostic subtypes (II, III, IV), and both patients experienced relapse. Of the 15 HR medulloblastomas, seven were classified as poor prognostic subtypes.

### Prognostic implication of molecular biomarkers in medulloblastoma

**Fig. 3:**
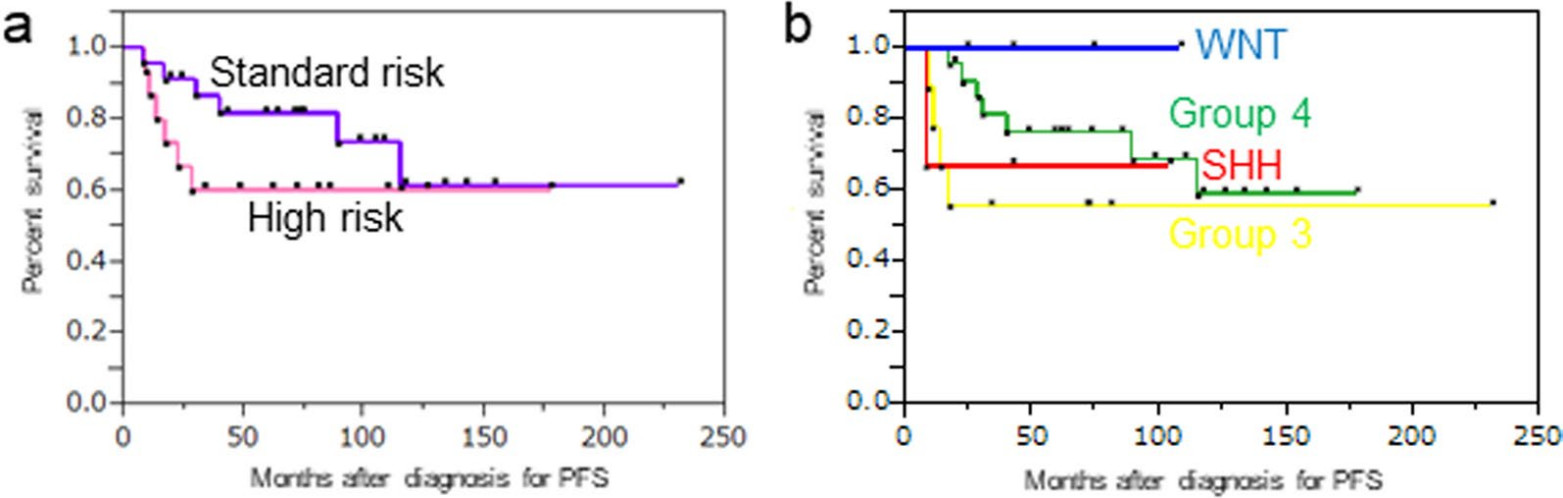
Progression-free survival of all patients stratified by **a** clinical risk and **b** molecular subgroup

**Fig. 4:**
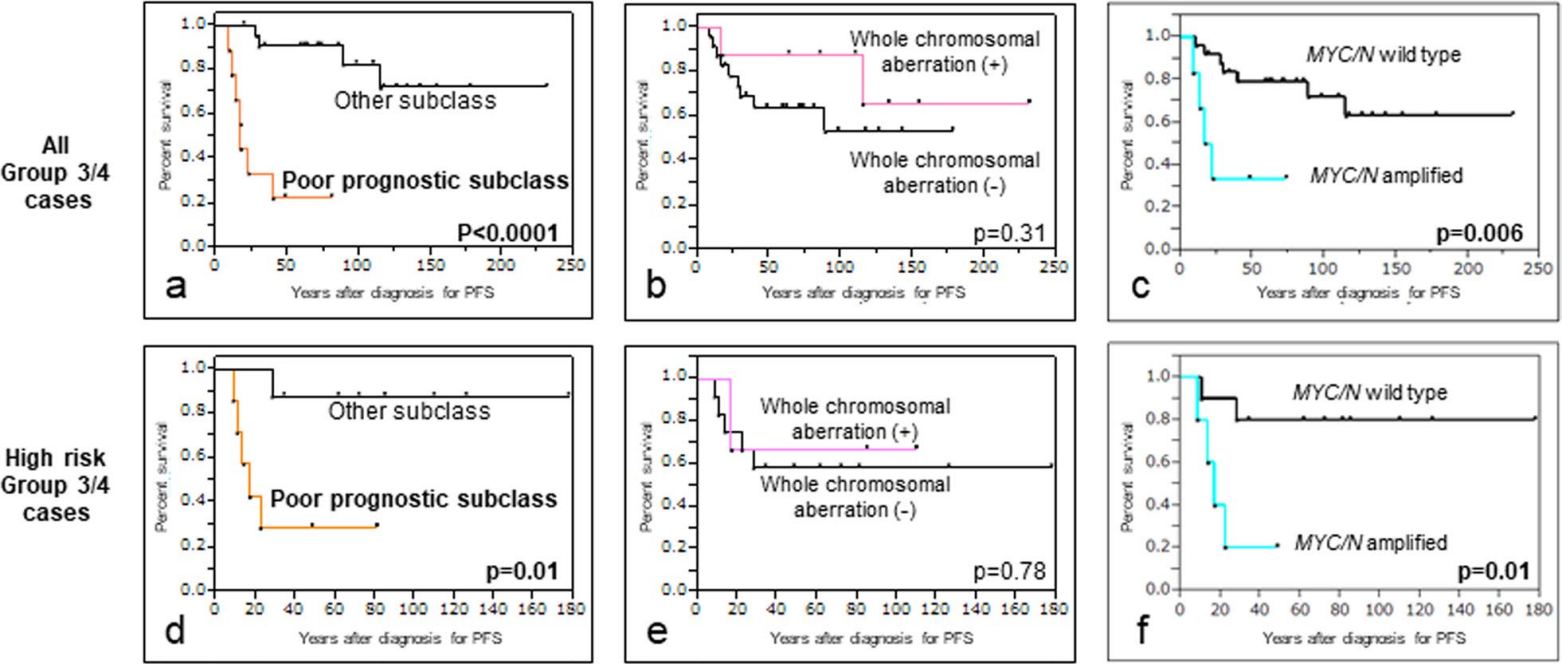
Progression-free survival of all patients with Group 3/4 medulloblastomas stratified by presence of poor prognostic subtyping (**a**), whole chromosomal aberration (**b**), and *MYC/N* amplification (**c**). Progression-free survival of high-risk patients with Group 3/4 medulloblastomas stratified by presence of poor prognostic subtyping (**d**), whole chromosomal aberration (**e**), and *MYC/N* amplification (**f**)

Prognostic analysis showed a 5-year progression free survival rate (5yPFS) of 81.7% in the SR group and 60.0% in the HR group (Fig. 3A). All recurrent cases displayed a distant relapse pattern after the completion of primary treatment (Additional file 1: Table). All WNT patients were alive without evidence of disease, and one of the SHH patients with *MYCN* amplification died of disease during the observation period. Regarding patients with Group 3/4 tumors, those with Group 3 tumors had the worst prognosis (5yPFS 55.5%) and those with Group 4 tumors showed intermediate survival (5yPFS 76.3%) (Fig. 3B). We then investigated the correlation between the prognosis of patients with Group 3/4 tumors and several previously reported molecular markers, including WCA, *MYC*/*MYCN* amplification, and poor prognostic subtypes. There were statistical prognostic differences in patients depending on the presence of *MYC*/*MYCN* amplification or poor prognostic subtypes in all Group 3/4 cases (Fig. 4A–C), and the result was reproducible in HR Group 3/4 cases (Fig. 4D–F). Only one recurrent case outside poor prognostic subtypes was present in our HR cohort, even when treated with lower dose CSI as 18.0 or 23.4 Gy than standard irradiation. In multivariate analysis, poor prognostic subtypes were only detected in both PFS and overall survival (OS) (Table 2, hazard ratio 12.77, 95% confidence interval (CI) for hazard ratio 2.38–99.21, *p* value 0.0026 for PFS, hazard ratio 5.02, 95% CI for hazard ratio 1.03–29.11, *p* value 0.044 for OS).

**Table 2:** Multivariate analysis of progression free survival (PFS) and overall survival (OS) among medulloblastomas

Variable	Hazard ratio (HR)	95% confidence interval for HR	*p* value
*Multivariate analysis of PFS among Group 3/4 medulloblastoma cases*			
Poor prognostic subclass	12.77	2.38–99.21	0.0026
*MYC/N* amplification	1.40	0.31–6.37	0.64
Whole chromosomal aberration	0.79	0.26–8.88	0.79
*Multivariate analysis of OS among Group 3/4 medulloblastoma cases*			
Poor prognostic subclass	5.02	1.03–29.11	0.044
*MYC/N* amplification	2.60	0.52–13.26	0.23
Whole chromosomal aberration	0.50	0.026–3.21	0.51

## Discussion

The treatment for medulloblastoma is multimodal, including surgery, chemotherapy, and focal irradiation to the tumor site, as well as CSI to prevent disseminated relapse. The CSI dose is stratified according to the presence of metastasis and resection rate at surgery. Thirty-six gray of CSI has been the mainstay of treatment for patients with metastatic tumors [13]. However, neurological sequelae caused by irradiation are one of the greatest issues to resolve. There are several reports on the correlation between CSI dose and neurocognitive decline after treatment [2, 3, 14, 15]. Mulhern described the results of the neurodevelopmental examination of patients enrolled in the SJMB96 clinical trial [14]. A statistical difference in intellectual decline was shown between patients who received 23.4 Gy and 36/39.6 Gy of CSI. In a large cohort of long-term acute lymphoblastic leukemia survivors, significant intelligence impairment was observed in patients who received 24 Gy of cranial irradiation (CRT), although few differences were noted between groups treated without CRT and those treated with 18 Gy CRT [3]. In a clinical trial involving patients aged 3 to 7 years with SR medulloblastoma, irradiation at 18.0 Gy as LD-CSI was found to lessen the decline in patients’ intelligence quotient, compared to that with 23.4 Gy as the standard-dose CSI. This finding indicated that the clinical impact of reducing the CSI dose may be beneficial not only for patients with metastatic tumors or large residues but also for SR patients. Moreover, the intellectual outcome after treatment may also depend on radiation modalities. A multi-institutional study by Kahalley et al. demonstrated that medulloblastoma patients treated with proton irradiation showed better neurocognitive results than those treated with conventional irradiation [17]. However, we are unable to comprehensively address this matter using our data because of the limited number of patients who underwent proton radiation therapy within our cohort.

To reduce the treatment burden, several studies have been conducted to identify prognostic biomarkers of medulloblastomas. WNT-activated subgroup medulloblastoma occurs in children, coincides with *CTNNB1* mutation, and shows the best prognosis among the tumors [1, 4]. Regarding copy number abnormalities, amplified *MYC* is frequently observed in Group 3 tumors, while *MYCN* amplification is common in the SHH subgroup [4, 5]. These amplifications serve as poor prognostic molecular markers [4], applying not only to all subgroups but also within each group [4, 5], which was reproducible from our data. In the clinical context, Detection of these alterations by fluorescence in situ hybridization or microarray can sometimes be difficult because of the spatial heterogeneity of the amplifications among tumor materials [5]. WCA is a copy number signature including chr 7 gain, chr 8 loss, and chr 11 loss to search for Group 3/4 medulloblastoma showing better clinical outcome. Goschzik et al. presented favorable prognostic outcomes of non-WNT/non-SHH medulloblastoma with WCA from a retrospective analysis of the HIT-SIOP PNET 4 trial cohort consisting of 338 patients [6]. Recently, subtyping of Group 3/4 medulloblastomas has been proposed by several institutions [7]. Researchers from several large institutions analyzed group 3/4 cases together and reported that they were separated into eight molecular subtypes based on DNA methylation profiles. Some subtypes include both groups of tumors because of the molecular ambiguity between them. Importantly, subtypes II, III, and V are clinically worse than the rest of the subtypes [7]. These poor prognostic subtypes may also be molecular markers for identifying patients that need more intensive treatment. Although prospective studies with a significantly larger cohort are necessary, the present study findings suggest that the novel subtyping may have potential as a prognostic biomarker, even among patients treated with LD-CSI. It is desirable for clinically high-risk Group 3/4 cases outside of the poor prognostic subtypes to reduce the CSI dose from 36 Gy, which leads to severe neurocognitive impairment. Thus, these findings support the expectation of treatment stratification based on subtyping, including metastatic tumors, in future clinical trials.

We acknowledge a limitation of this study; we were unable to provide quality of life assessment data or neurocognitive functional testing data from our cases. Future trials aiming to validate our findings should incorporate prospective intelligence assessments of registered cases because objective data are essential to support the benefits of reduced irradiation.

Last, caution should be exercised with regard to late recurrence in “SR” medulloblastomas. It is known that some group 4 tumors recur very late, that is, after 5 years from onset. In our cohort, there were two late recurrences and one of them was subtype VII, which was not included in the poor prognostic subtype [16]. This very late recurrent case indicates a limitation of the novel subtyping; thus, efforts to enable much better risk stratification to improve the prognosis and quality of life of patients with medulloblastoma should continue.

## Conclusions

Molecular subtyping of medulloblastomas may be a potential factor for stratifying Group 3/4 tumors in the treatment of patients with metastatic medulloblastomas that have never undergone prognostic stratification.

### Supplementary Information

**Additional file 1.** Supplementary table.

## Data Availability

The data that support the findings of this study are available from the corresponding author upon reasonable request.
